# Online sperm donation: a survey of the demographic characteristics, motivations, preferences and experiences of sperm donors on a connection website

**DOI:** 10.1093/humrep/dew166

**Published:** 2016-08-19

**Authors:** T. Freeman, V. Jadva, E. Tranfield, S. Golombok

**Affiliations:** 1 Centre forFamily Research, Department of Psychology, University of Cambridge, Free School Lane, Cambridge CB2 3RF, UK; 2 Pride Angel, www.prideangel.com

**Keywords:** sperm donor, online connection website, donor conception, anonymous, open-identity and known donation, sexual orientation

## Abstract

**STUDY QUESTION:**

What are the demographic characteristics, motivations, preferences and experiences of heterosexual, gay and bisexual sperm donors on a connection website (i.e. a website that facilitates direct contact between donors and recipients of gametes)?

**SUMMARY ANSWER:**

This demographically diverse group of men was donating for altruistic reasons and perceived the website as providing greater choice over donation arrangements: approximately one third favoured anonymous donation, most of whom were heterosexual, whilst gay and bisexual donors were more likely to be in contact with children conceived with their sperm.

**WHAT IS KNOWN ALREADY:**

Despite substantially more sperm donors being registered on connection websites than with clinics, there has been very little research on this population. Current understanding of the impact of sexual orientation on donors' attitudes is also limited.

**STUDY DESIGN, SIZE, DURATION:**

An online survey was conducted over 7 weeks with 383 men registered as sperm donors with Pride Angel, a large UK-based connection website for donors and recipients of sperm.

**PARTICIPANTS/MATERIALS, SETTING, METHODS:**

The survey obtained data on participants' demographic characteristics and their motivations, preferences and experiences regarding online sperm donation, including attitudes towards contact with offspring. Differences according to participants' sexual orientation were examined.

**MAIN RESULTS AND THE ROLE OF CHANCE:**

Most participants (80.4%, 308) were heterosexual, 10.5% (40) were gay and 9.1% (35) were bisexual; ages ranged from 18 to 69 years (median = 36, mean = 37.3, SD = 9.7). A greater proportion of gay and bisexual men desired open-identity donation (*P* < 0.005) and contact with offspring (*P <*0.005) than heterosexual men. Approximately one third (28.7%, 110) had donated sperm; 18.3% (70) had conceived at least one child, of whom a minority (25.7%, 18) were currently in contact with the child, comprising significantly more gay and bisexual than heterosexual men (*P* = 0.001). Heterosexual men were most likely to state a preference for natural insemination, although the large majority (94.3%, 66) of donors who had conceived children had used artificial insemination.

**LIMITATIONS, REASONS FOR CAUTION:**

Findings may not be representative of all sperm donors using connection websites because members of only one website participated and participants were, by necessity, a self-selected sample.

**WIDER IMPLICATIONS OF THE FINDINGS:**

This is the first comprehensive study of donors who connect with recipients via the internet, including a substantial number who have donated and conceived children. The findings indicate that sexual orientation may influence men's donation preferences and raise policy issues concerning donor recruitment and the incorporation of online sperm donation into clinical practice.

**STUDY FUNDING/COMPETING INTEREST(S):**

This study was supported by the Wellcome Trust (097857/Z/11/Z). E.T. is the co-founder of Pride Angel; the remaining authors have no conflicts of interest.

## Introduction

In recent years, there has been a sharp growth in the number of men seeking to become sperm donors via connection websites (websites designed to facilitate contact between those wishing to donate and receive gametes) rather than through the regulated routes of fertility clinics and sperm banks (Human Fertilisation and Embryology Authority, 2014a). Although the practice of informal donation long precedes the rise of connection websites, this development has made non-regulated routes to sperm donation more accessible. Possible reasons for this trend include the rise of online social networking, the high expense of fertility treatment, growing numbers of single women and lesbian couples seeking donor sperm and the introduction of open-identity donation in several countries. In the UK, donor anonymity was removed in 2005 so all who donate through clinics now agree to their identity being accessible to donor-conceived individuals at age 18 years. Open-identity donation may increase donors' desire for control over who receives their gametes (Pennings, 1995); likewise some recipients may seek more choice and information about who their donors are and what the conditions of the donation should be (Freeman *et al*., 2012).

Online sperm donation raises concerns about personal, medical and legal risks of private donation arrangements without the regulatory protection of a licensed clinic. For example, the UK's regulatory body, the Human Fertilisation and Embryology Authority (HFEA) highlights concerns about the resultant child's legal parentage and future access to donor information, the number of children created from any one donor, the lack of donors' medical screening and the potential sexual exploitation of recipients (Human Fertilisation and Embryology Authority, 2014a). Despite such concerns, very little is presently known about online sperm donors. Research has principally focused on the motivations of clinic donors, which are generally reported as a combination of altruism and, where applicable, financial compensation (Daniels *et al*., 2005; Ernst *et al*., 2007; Frith *et al*., 2007; Bay *et al*., 2014). Secondary motivations such as the desire to procreate, to check one's fertility and the reciprocity of those undergoing fertility treatment are also sometimes found (Riggs and Russell, 2011; Jadva *et al*., 2011). Specific reasons for choosing to donate in a non-clinic context have not been examined, with the exception of two small scale Dutch studies (Bossema *et al*., 2014; Woesternburg *et al*., 2015). For example, Bossema *et al*.'s (2014) study included five sperm donors who had donated in an ‘informal setting’, finding their reasons for this preference included engagement in the process and the potential to have a bond with the recipient and contact with the child.

More recently, attention has turned to the changing demographic characteristics and attitudes of men who are willing to donate and to be identifiable; the common view being that the removal of donor anonymity initiated a shift from younger single donors motivated by financial compensation to older married men donating for altruistic reasons (Van den Broeck *et al*., 2013). Despite a general preference for anonymity amongst prospective donors (Godmand *et al*., 2006), several studies indicate that some are willing to be identifiable (Lalos *et al.,* 2003; Daniels, 2007; Frith *et al*., 2007) and that this may vary by demographic background (Van den Broeck *et al*., 2013). In particular, a man's age, marital status and parental status have been highlighted as impacting on attitudes towards open-identity donation: for example, being in a relationship and having children have been associated with men being less inclined to meet their donor offspring (Godmand *et al.,* 2006; Riggs and Russell, 2011).

Recent research indicates that sexual orientation may also be significant in understanding donors' motivations and attitudes towards donation (Riggs, 2008). For example, an Australian survey of online donor profiles found that men in same-sex relationships would be more likely to consent to identity-release donation than those in heterosexual relationships (Riggs and Russell, 2011). However, whilst details of a donor's age, marital status and parental status are routinely collected by HFEA licenced clinics, information about their sexual orientation is not; likewise most studies of donors' characteristics do not report their sexual orientation (e.g. Sydsjo *et al*., 2012). Furthermore, although many UK clinics openly recruit gay men as donors, others do not because of the perceived link between homosexuality and sexually transmitted diseases such as HIV (National Gamete Donation Trust, 2015).

There has been very little research on men's experiences of donation and their attitudes towards the resulting children. Some studies indicate men's increased openness to contact with donor offspring after donating (Daniels *et al*., 2005; Ernst *et al*., 2007), although information about the frequency and nature of desired contact, and whether this is achieved in practice, has not been reported. Furthermore, in their systematic review, Van den Broeck *et al*. (2013) found the proportion of actual donors who wished for contact with donor offspring varied greatly across studies, with demographic factors again partly explaining this difference. Further research addressing both potential and actual donors' attitudes towards donor offspring is therefore needed.

This is the first large-scale survey of men registered as online sperm donors and also, of UK sperm donors since donor anonymity was removed. The study aimed to examine the demographic backgrounds, motivations and preferences of both potential and actual sperm donors registered with Pride Angel (www.prideangel.com); a UK-based connection website that was selected for study as one of the largest and well known of its kind. Key questions to be addressed include: Who are these sperm donors? What motivates them to donate their sperm and why are they using a connection website to do so? What method of donation do they intend to use? What have they done in preparation for donating? What are their expectations regarding contact with recipient families? How many have actually donated sperm? Do they keep in touch with the families they help create? As existing research suggests that sexual orientation may influence men's views on donation (Riggs, 2008), findings have been examined in relation to this variable.

## Materials and Methods

All Pride Angel members were sent an email invitation from the founder of Pride Angel (E.T.) containing a link to the survey and consent procedures, followed up where applicable by two reminder emails. Information about the study was also advertised on the Pride Angel home page. The survey was live for 7 weeks during February–March 2014. Participants received 10 free message credits (current value £10) on completing the survey.

At the survey start, online membership (i.e. those with web profiles) of Pride Angel was 27 650 persons, of whom 5299 (19.2%) were registered as sperm donors; there were also 17 367 (62.8%) registered sperm recipients, 547 (2.0%) registered egg donors, 866 (3.1%) registered egg recipients and 3571 (12.9%) registered co-parents (see Jadva *et al*. (2015) for a report on co-parents' data). Of a total of 32 634 invitations emailed to all members (i.e. including those without web profiles), 5425 were opened, representing 19.6% of online members and 16.6% of all members. Of those who opened the email, 1402 (25.8%) started the survey and 1022 (18.8%) completed it. A total of 400 registered sperm donors completed the survey, comprising 38.5% of the estimated number (i.e. 1040) who opened the email. Seventeen participants were excluded from analysis because they did not identify as ‘heterosexual’, ‘gay’ or ‘bisexual’, giving a sample size of *n* = 383.

### Measures

The survey comprised multiple choice and open-ended questions. For all participants, data were obtained on (i) Socio-demographic characteristics: sexual orientation, age, relationship status, parental status, country of residence, ethnicity, education, employment status, (ii) Motivations for seeking to donate sperm, (iii) Motivations for using a connection website, (iv) Preferences regarding sperm donation arrangements: type and method of donation, contact with donor offspring, (v) Preparations for donation: telling partner, legal, psychological and medical preparations, (vi) Experience of donation: donating elsewhere, outcome of using connection website. Additional questions for actual sperm donors (i.e. whose donations via Pride Angel had led to at least one child) included (vii) Method of donation used, (viii) Number and age of children born, (ix) Nature and frequency of contact with child. Where relevant, question wording was in line with terminology used by connection websites (e.g. response options regarding preferred method of donation included terms ‘artificial insemination’ and ‘natural insemination’).

### Statistical analyses

Data are presented first for the whole sample (i.e. ‘all donors’) and second, for the subsample who had conceived children as sperm donors via Pride Angel (‘actual donors’). Comparisons were conducted by sexual orientation (heterosexual versus gay and bisexual) using Chi-square and Fisher's exact tests of significance at the 0.05 level for all variables except motivations for donating, for which Mann–Whitney *U*-tests were used. Responses to an open-ended question about donors' motivations for registering on the connection website were systematically categorized into themes using Atlas-ti v7 (GmbH, Berlin). As not all participants answered each question, the analyses only include those who responded.

### Ethical approval

Ethical approval for this study was obtained from the University of Cambridge Psychology Research Ethics Committee.

## Results

### All sperm donors

#### Characteristics of all sperm donors

Eighty per cent (308) of participants were heterosexual, 10.5% (40) were gay and 9.1% (35) were bisexual. Approximately half reported being single (52.5%, 201) and not having children (47.3%, 181), with a significant difference by sexual orientation: 55.5% (171) of heterosexual men were single compared with 40.0% (30) of gay and bisexual men (*χ*^2^ (1, *n* = 381) = 5.50, *P <*0.05) and 61.3% (46) of gay and bisexual men reported not having children compared with 43.8% (135) of heterosexual men (*χ*^2^ (1, *n* = 381) = 7.16, *P* < 0.01). Further socio-demographic characteristics of all sperm donors are given in Table I, with no significant differences by sexual orientation.

**Table I:** Socio-demographic characteristics of all donors and ‘actual donors’.*

	All donors		Actual donors	
	Mean	SD	Mean	SD
Age	37.3	9.7	38.69	8.41
Age range (min-max)	18–63 years		23–60 years	
	***n***	**%**	***n***	**%**
Country of residence				
United Kingdom	156	40.7	50	71.4
United States	59	15.4	5	7.1
India	48	12.5	2	2.9
Canada	27	7.0	5	7.1
Australia	19	5.0	5	7.1
Ireland	6	1.6	0	0
New Zealand	6	1.6	0	0
Hungary	5	1.3	1	1.4
South Africa	5	1.3	1	1.4
The Netherlands	3	0.8	1	1.4
Other**	49	12.8	0	0
Ethnicity				
White	254	66.3	62	88.6
Asian	85	22.2	3	4.3
Black	28	7.3	3	4.3
Mixed race	12	3.1	2	2.9
Other	4	1.0	0	0
Education				
Less than secondary school	3	0.8	3	4.3
Secondary school	39	10.2	3	4.3
College or trade qualification	115	30.0	22	31.4
University degree or higher	226	59.0	42	60.0
Employment status				
Employed full-time	266	69.5	60	85.7
Employed part-time	54	14.1	4	5.7
Not employed	59	15.4	5	7.1
*Not specified*	4	1.0	1	1.4

*‘Actual donors’ refers to men whose donations via Pride Angel had led to the live birth of at least one child.

**Includes Bahrain, Bangladesh, Brazil, China, Croatia, Denmark, France, Germany, Ghana, Indonesia, Israel, Kenya, Lebanon, Malaysia, Malta, Nigeria, Oman, Pakistan, Portugal, Singapore, Slovenia, Spain, Sri Lanka, Ukraine, United Arab Emirates, Zambia (≤1.3%, 5 in each).

#### Motivations for donating sperm and for using a connection website

Participants were asked to rate the importance of motivations for donating sperm (Table II). The highest rated were altruistic (e.g. ‘want to help others’), then procreative (e.g. ‘to pass on my genes’), followed by motivations relating to personal experience or circumstance (e.g. ‘family/friends have experienced infertility’). ‘Financial payment’ was regarded least important, with 49.6% (114) rating this as ‘not important at all’. The only significant differences for motivations by sexual orientation were for ‘want to help others’ (*H* = 6.08, df = 1, *P* = 0.014) and ‘no reason not to’ (*H* = 4.65, df = 1, *P* = 0.031) which heterosexual men tended to rate more highly than gay and bisexual men. As reported in Table III, six themes were identified regarding motivations for using a connection website.

**Table II:** Motivations to donate sperm for all sperm donors by sexual orientation.

	Sexual orientation				Total**		
	Heterosexual		Gay and bisexual				
Motivations*	Median (interquartile range)	*n*	Median (interquartile range)	*n*	*P*	Median (interquartile range)	*n*
Want to help others	5 (0)	299	5 (1)	71	0.014	5 (0)	370
To do something valuable and worthwhile	5 (1)	287	5 (1)	69	ns	5 (1)	356
To enable others to enjoy parenting as I have myself	5 (1)	230	5 (1)	43	ns	5 (1)	273
I do not have a partner to have children with	3 (2)	232	3 (2)	55	ns	5 (3.5)	201
To pass on my genes	4 (2)	265	4 (2)	62	ns	4 (2)	327
To have children/procreate	4 (2)	260	4 (2)	66	ns	4 (2)	326
My sperm would go to waste otherwise	4 (2)	248	4 (3)	61	ns	4 (2)	309
I don't want to have children myself	3 (3)	179	3 (3)	49	ns	3 (3)	228
Confirmation of my own fertility	3 (3)	222	2 (4)	60	ns	3 (3)	282
Family/friends have experienced infertility	3 (2)	142	3 (3)	36	ns	3 (3)	178
Family/friends have used sperm or egg donation	3 (3)	121	3 (3)	39	ns	3 (3)	178
My partner is infertile or has fertility problems	3 (3)	99	2 (3)	27	ns	3 (3)	126
No reason not to	3 (2)	232	3 (2)	55	0.031	3 (2)	287
I am single	3 (2)	176	3 (4)	39	ns	3 (2)	215
Financial payment	2 (2)	182	1 (2)	48	ns	2 (2)	230
Other reason	3 (2)	38	5 (2)	15	ns	3 (2)	53

*Scale ranged from 1 ‘not at all important’ to 5 ‘very important’.

**Sample size comprises number of respondents who ranked each motivation.

**Table III:** Reasons for using a connection website.

Theme*	Description	Quotations
Attributes of website (*n* = 79)	Positive reputation and qualities	‘*serious’, ‘trustworthy’, ‘moderated’, ‘safe’, ‘popular’*
Access to recipients (*n* = 26)	Including access to recipients in genuine need, the LGBT community, countries where sperm donation services are limited/prohibited	*‘Access to truly interested people’* *‘Reaches those who cannot afford private treatment’*
Ease of use (*n* = 17)	Directness of website as medium of contact	*‘It was simple, straightforward, and flexible—it was really up to me how it would progress, so I could always stop if not happy’* *‘It acted as a medium for two parties to connect—no real middlemen, or hoops to jump through’*
Communication and contact (*n* = 12)	Enables communication with recipient before and after birth and contact with child	*‘I'd like to have some connection with the recipient, to know it all worked out well’* *‘Allows me to have contact with the child before he or she is 18 years old’*
Control and choice (*n* = 9)	Including choice over recipients, level of communication with recipient families	*‘I have control over my anonymity in a way that I wouldn't through a clinic’* *‘Being able to find out a little bit about the prospective parents and retain some control on how my sperm is used’*
Other limitations of clinics (*n* = 5)	Including high costs, bureaucracy, regulations	*‘No red tape of clinic’* *‘Too old to donate at a sperm bank’*

*Coded from 134 responses to the open-ended question, ‘Why have you decided to donate your sperm through Pride Angel?’; responses that identified generic reasons for donating sperm rather than specific reasons for donating via a website were excluded.

#### Preferences regarding sperm donation arrangements

*Type and method of donation:* Approximately one third (31.5%, 118) of participants expressed a preference for anonymous donation, whilst over half (57.6%, 216) preferred some type of non-anonymous arrangement (i.e. identity release, known, co-parent, other). A greater proportion of heterosexual men stated a preference for anonymous donation than gay and bisexual men (*χ*^2^ (1, *n* = 334) = 10.31, *P <*0.005), for whom the majority (69.8%, 51) preferred a non-anonymous arrangement. The most common preferred method of donation overall was ‘natural insemination’ (44.1%, 164). There was a significant difference by sexual orientation, with proportionately more gay and bisexual men preferring donation ‘at a clinic’ than heterosexual men, of whom approximately half (48.3%, 145) reported ‘natural insemination’ as their preference (Fisher's exact = 0.000) (Table IV).

**Table IV:** Preferences regarding sperm donation arrangements for all sperm donors by sexual orientation.

	Sexual orientation					Total	
	Heterosexual		Gay and bisexual				
	*n*	%	*n*	%	*P*	*n*	%
Type of donation					<0.005		
Anonymous	107	35.4	11	15.1		118	31.5
Identity release	65	21.5	14	19.2		79	21.1
Known	78	25.8	32	43.8		110	29.3
Co-parent	18	6.0	5	6.8		23	6.1
Other non-anonymous	4	1.3	0	0		4	1.1
Other*/don't know	30	9.9	11	15.1		41	10.9
Method of donation					0.000		
Natural insemination	145	48.3	19	26.4		164	44.1
Artificial insemination	94	31.3	23	31.9		117	31.5
At a clinic	49	16.3	30	41.7		79	21.2
Other*	12	4.0	0	0		12	3.2
Meet child					<0.005		
Yes	109	36.2	43	58.1		152	40.5
Maybe	118	39.2	24	32.4		142	37.9
No	74	24.6	7	9.5		81	21.6
Contact with child					<0.005		
Yes	93	30.6	37	50.0		130	34.4
Maybe	124	40.8	27	36.5		151	39.9
No	87	28.6	10	13.5		97	25.7

*Includes ‘dependent on recipient's wishes’.

*Contact with donor offspring:* A minority of participants did not wish to meet (21.6%, 81) or be in contact with (25.7%, 97) their donor offspring. A greater proportion of gay and bisexual men reported wishing to meet (*χ*^2^ (2, *n* = 375) = 14.04, *P* < 0.005) and to be in contact with (*χ*^2^ (2, *n* = 378) = 12.09, *P*< 0.005) their donor offspring than heterosexual men (Table IV).

#### Preparation for sperm donation

*Telling partners:* Under half (45.0%, 81) of those currently in a relationship had discussed their plans to donate with their partners. Gay and bisexual men were more likely to have done so than heterosexual men (*χ*^2^ (1, *n* = 180) = 4.67, *P* < 0.05), of whom the majority (59.6%, 81) had not told their partners.

#### Experience of donation

*Alternative routes to donation:* A substantial proportion (41.5%, 159) of participants reported previously donating sperm elsewhere: 23.8% (91) via another connection website, 8.9% (34) via a clinic, 5.7% (22) via a sperm bank, 10.4% (40) to a friend and 1.0% (4) to a family member. A greater proportion of heterosexual men (45.6%, 139) had donated sperm previously than had gay and bisexual men (27.4%, 20) (*χ*^2^ (1, *n* = 378) = 7.99, *P <*0*.*01), although there were no significant differences by sexual orientation regarding routes to previous donations.

*Outcome of using connection website:* Approximately one half (48.8%, 187) of participants had made contact with at least one potential recipient via Pride Angel and 27.9% (107) had met one face-to-face; 28.7% (110) reported providing sperm to a recipient, of whom 15.5% (17) were unaware of the outcome. Overall 18.3% (70) of participants reported at least one child born from their donations via Pride Angel. There were no significant differences by sexual orientation regarding the number of participants who made contact with, or provided sperm to, recipients, or who had conceived children. The number of children born per donor ranged from 1 to 10 (median = 3), with 153 donor offspring reported in total. Almost 60% (57.1%, 40) of actual donors had conceived three or less children; 11.4% (8) did not know how many children had been born. Current age of eldest child born was 0–9 years (mean = 5.87, SD = 1.78).

### Actual sperm donors

#### Characteristics of actual sperm donors

Most donors who had conceived children were heterosexual (82.9%; 58), 11.4% (8) were gay and 5.7% (4) bisexual. Half (50.0%, 35) were single and a quarter (25.7%, 18) reported having no children. Further socio-demographic characteristics of actual donors are given in Table I, with no significant differences by sexual orientation. A greater proportion of actual donors were UK residents, white (Fisher's exact = 0.000), in employment (*χ*^2^ (1, *n* = 379) = 11.36, *P*< 0.005) and reported having children of their own (*χ*^2^ (1, *n* = 381) = 16.33, *P*< 0.001) compared with potential donors.

#### Preparation for sperm donation

*Legal, medical and psychological:* Most actual donors had undergone medical screening (87.1%, 61) and drawn up a legal agreement (52.9%, 37), 30.0% (21) had taken legal advice and 21.4% (15) had undergone counselling, with no significant differences by sexual orientation.

#### Sperm donation arrangements in practice

*Method of donation*: The large majority (94.3%, 66) of actual donors had donated by artificial insemination, 32.9% (23) by natural insemination and 5.7% (4) at a clinic, with no significant differences by sexual orientation. The responses indicate that some donors had donated on more than one occasion using more than one method.

*Contact with recipient families:* Most (72.9%, 51) actual donors had seen a photograph of the child conceived from their donation and a quarter (25.7%, 18) had met them and were currently in contact with them. More gay and bisexual donors (58.3%, 7) had met their donor offspring compared with heterosexual donors (19.0%, 11) (Fisher's exact = 0.009); likewise, more gay and bisexual donors (66.7%, 8) were currently in contact with the child compared with heterosexual donors (17.2%, 10) (Fisher's exact = 0.001). Gay and bisexual men were in more frequent contact (Fisher's exact = 0.000) with donor offspring than heterosexual men who were most likely to have no contact, although it should be noted that the numbers in this subgroup of donors were low (Table V).

**Table V:** Actual donors' frequency of contact with donor offspring* by sexual orientation.

	Sexual orientation				Total	
	Heterosexual		Gay and Bisexual			
	*n*	%	*n*	%	*n*	%
**Frequent**	**2**	**3.4**	**7**	**58.3**	**9**	**12.9**
Everyday	0	0	1	8.3	1	1.4
Once a week	1	1.7	1	8.3	2	2.9
Once a fortnight	0	0	1	8.3	1	1.4
Once every 1–2 months	1	1.7	4	33.3	5	7.1
**Occasional**	**12**	**20.7**	**0**	**0**	**12**	**17.1**
Once every six months	5	8.6	0	0	5	7.1
Once a year	4	6.9	0	0	4	5.7
Less than once a year	3	5.2	0	0	3	4.3
**No contact**	**44**	**75.9**	**5**	**41.7**	**49**	**70.0**

*Participants with >1 donor offspring referred to the child they had most contact with.

Bold values indicate significance at *P* = 0.00.

## Discussion

This study indicates that online sperm donors form a demographically diverse group with primarily altruistic motivations for donating. These donors varied in their attitudes towards donation, with marked differences arising according to sexual orientation. Gay and bisexual men expressed a preference for open-identity donation and were more likely to be in contact with children conceived with their donated sperm, whilst heterosexual men more frequently sought anonymous donation. The website was perceived as facilitating these different goals by allowing greater choice and control over the donation process than clinics. Heterosexual men were also more likely to favour natural insemination compared with the gay and bisexual group who preferred donation at a clinic, although the vast majority of men who conceived children had used artificial insemination in practice.

The survey provides new information about the nature of online sperm donation and raises wider policy issues. Regarding demographic characteristics, there was a relatively high proportion of heterosexual donors given the website's open orientation towards the lesbian, gay, bisexual, and transgender (LGBT) community. Also notable was that approximately one third were over the HFEA's recommended maximum age of 40 years for UK sperm donors, although this limit is discretionary and recent figures suggest an increase in older clinic donors (Human Fertilisation and Embryology Authority, 2014b). In addition, approximately one fifth classified their ethnicity as Asian and many (12.5%) lived in India. This is pertinent to UK practice given concerns about the lack of sperm donors from minority ethnic groups (Nuffield Council on Bioethics, 2013). Furthermore, the sample's wide geographical spread across 36 countries raises legal and ethical issues concerning new possibilities for transnational reproduction. Despite this variation, the majority of actual donors were white UK residents. This reflects the demographics of sperm recipients on Pride Angel of whom the majority are also white UK residents, although further analyses is required to ascertain the extent to which recipient parents seek donors with similar characteristics to their own.

Regarding motivations, the finding that the majority of these online donors were pursuing donation for altruistic reasons accords with existing research on clinic donors. Procreative motivations were also identified as important, as found in Woesternburg *et al*.'s (2015) study of online sperm donors. This study also confirms that men's specific reasons for registering with websites rather than clinics include the greater potential to engage with recipient families (Bossema *et al.,* 2014; Woesternburg *et al.,* 2015). However, the current study goes further in demonstrating the diversity of donors' preferences, giving a broader picture of online sperm donation overall. Most strikingly, a sizeable minority pursued online donation to facilitate their anonymity and minimal contact with recipient families. The meaning of anonymity in this context requires further investigation: as well as referring to anonymity from the child, donors and recipients may make practical arrangements to conceal the donor's identity from the recipient as well. Clearly the use of websites for anonymous donation raises regulatory issues given that this is prohibited in the UK and elsewhere. Conversely, the greater openness to open-identity donation amongst gay and bisexual men has wider implications for practice, including whether information about donors' sexual orientation should be recorded and different recipient groups' attitudes towards gay donors.

A further salient finding was the heterosexual men's stated preference for natural insemination. However, the high number who used artificial insemination in practice reflects not only that Pride Angel seeks to prohibit members from pursuing natural insemination but also that recipients may be ‘filtering out’ donors who are less well-intentioned; indeed, those donors who conceived children appeared a responsible group, with many having untaken medical and legal preparations for donation. Heterosexual donors were also less likely to discuss their donation plans with their partners. Whilst previous research indicates that only a minority of clinic donors involve their partners in the decision-making process (Van den Broeck *et al*., 2013), it may be that the medium of the internet both enables and encourages secrecy. A further regulatory issue is that many men had donated previously elsewhere, including via other websites, clinics and sperm banks. This has important ramifications for controlling offspring numbers. Within this sample, the largest number of children born per donor was 10 which is within the current UK limit of 10 families per donor. However, their donations via other routes may have led to further pregnancies; furthermore, a small minority were unware of the amount of children born.

There are important limitations to acknowledge when interpreting these findings. Although data were collected from a large sample of donors, findings may not be representative of all sperm donors on connection websites as members of only one website participated and the sample was by necessity self-selected. Furthermore, response rates in online surveys are typically low and difficult to calculate (Hewson, 2014), although this method also has advantages including reaching large numbers from hard-to-access populations (Wright, 2005). Indeed, as an active online community, Pride Angel lends itself to web-based research and the sample size achieved here was large compared with other studies of sperm donors, allowing for within-group comparisons. A comparison of study participants with the total population of sperm donors on Pride Angel found this sample to be comparable in terms of sexual orientation (study participants: 80.4% heterosexual, 10.5% gay, 9.1% bisexual cf. Pride Angel members: 78.8% heterosexual, 14.1% gay, 7.1% bisexual), although further comparisons could not be made because Pride Angel members are not required to submit demographic information.

Despite these limitations, this study provides some valuable empirical insights. In particular, the potential impact of sexual orientation on donors' preferences merits further investigation. Previous research highlights the importance of distinguishing between donors' willingness to be identifiable and to be contacted by donor offspring (Godmand *et al*., 2006). The finding that gay and bisexual men were not only favourable to open-identity donation but wished for contact with recipient families is therefore illuminating and may indicate a view of sperm donation as embracing more involved ‘parental’ aspects of procreation; this appears to be reflected in practice by the greater frequency of contact with donor offspring amongst the gay and bisexual group. An overall finding is that, despite the concerns raised, online sperm donation is being utilized in large numbers: within this sample, 70 men had successfully donated and helped create over 150 children. Furthermore, a small proportion of these donations had occurred in clinics and an even greater number of donors wished for clinic donation, particularly gay and bisexual men. This demonstrates that the distinction between ‘clinic’ and ‘online’ sperm donation is being blurred in practice and highlights the importance of considering ways that online donors may be further incorporated into clinic treatments.

## Authors' roles

All authors were involved in designing this study. E.T. assisted with the recruitment of participants. All other authors were involved in the analysis and interpretation of data. This manuscript was drafted by T.F. and has been approved by all authors.

## Funding

This study was supported by the Wellcome Trust (097857/Z/11/Z). Funding to pay the Open Access publication charges for this article was provided by the University of Cambridge.

## Conflict of interest

E.T. is Director and Co-Founder of the website www.prideangel.com. The other authors have no conflicts of interest to declare.
